# Temporal Aspects of Memory: A Comparison of Memory Performance, Processing Speed and Time Estimation Between Young and Older Adults

**DOI:** 10.3389/fnagi.2018.00352

**Published:** 2018-11-06

**Authors:** Deena Ebaid, Sheila G. Crewther

**Affiliations:** Department of Psychology and Counselling, School of Psychology and Public Health, La Trobe University, Melbourne, VIC, Australia

**Keywords:** memory, processing speed, aging, cognition, retrospective time estimation, time estimation, temporal estimation

## Abstract

Cognitive abilities are often reported to decline across the lifespan, particularly when assessed with working memory (WM) measures such as the auditory backward digit span and complex *N-*back tasks. However, some debate still exists regarding which aspects of cognition are most susceptible to the aging process and which may remain intact. Additionally, time estimation, though a complex psychological dimension, is often studied in relative isolation and is particularly neglected in traditional studies of WM, with little research from the viewpoint of retrospective temporal estimation. In particular, research seldom considers whether the ability to accurately estimate time retrospectively, is correlated with performance on traditional memory and processing speed measures in healthy populations. Thus, we chose to investigate performance of comparably educated young and older adult groups on both classical memory tasks including auditory and visual digit spans, *N*-back, Wechsler Adult Intelligence Scale (WAIS)-based measures of processing speed (i.e., Symbol Search [SS] and Coding [Cod]) and a temporal measure of WM with a focus on retrospective time estimation. Our sample included 66 university students (58 F, 8 M) between the ages of 18–29, and 33 university-educated healthy older adults (25 F, 8 M) between the ages of 60–81. Results indicated that older adults performed significantly worse on auditory but not the visual digit span tasks, as well as on both the SS and Cod, though performed equally well on the *N* = 1 back task. Results also showed that retrospective time estimation was not significantly different between young and older adults, with both groups substantially underestimating duration of a simple task. Retrospective time estimation was not significantly correlated to any memory or processing speed measure, emphasizing the need for future research into the specific cognitive domains underlying the subjective estimation of a temporal interval.

## Introduction

Aging is often associated with a decline in a range of cognitive abilities relating to measures of working memory (WM), dual tasks and executive functioning (Salthouse, 1990; Moore et al., 2005; Gazzaley et al., 2007; Wang et al., 2011; Kirova et al., 2015; Kato et al., 2016; Vellage et al., 2016). Traditionally, WM is defined as the memory system involved in actively maintaining current information for a period of time, allowing for on-line or later manipulation and access, and is suggested to underpin complex processes such as learning, reasoning, planning and problem solving (Baddeley and Hitch, 1974; Baddeley, 1986, 2007; Baddeley et al., 2009). WM is also considered to be limited in the number of items that can be temporarily stored and manipulated simultaneously (Baddeley and Hitch, 1974; Salthouse, 1990; Baddeley, 2007) presumably due to limited attention span and capacity over a particular period of time (Cowan, 1998; Wiley and Jarosz, 2012). Interestingly, few reports have considered the aspects of WM that are optimum for the accurate perception of time in healthy older populations. Variables such as sensory (vision and hearing) receptor integrity (Lindenberger and Baltes, 1994; Baltes and Lindenberger, 1997; Füllgrabe et al., 2015), education and affective factors have been suggested to contribute to the decline in WM seen with age (Hester et al., 2004; Beaudreau and O’Hara, 2008, 2009; Hammar and Årdal, 2009; Puccioni and Vallesi, 2012; Vallesi, 2016). Specifically, more years of formal education are suggested to mitigate cognitive decline seen in aging, while higher depressive and anxiety symptoms are consistently reported to impede on cognitive performance in all populations (Beaudreau and O’Hara, 2008, 2009; Hammar and Årdal, 2009).

Commonly used experimental measures of processing speed which often show decline across the lifespan are the Symbol Search (SS) and Coding (Cod) tasks from the Information Processing Speed Index of the Wechsler Adult Intelligence Scale (WAIS; Wechsler, 2008a,b) i.e., Cornelis et al. (2015); Joy et al. (2004). In addition, one of the simplest and most commonly used WM measures is the *N-*back task, which involves presenting participants with a series of stimuli (predominantly visual) requiring a manual response to a nominated target stimulus that had been presented *N* items earlier (Kirchner, 1958). Research has commonly reported that older adults perform worse at this task when *N* = 2 or more and are more susceptible to distractors than younger adults (i.e., Kato et al., 2016). Neuroimaging data has linked good performance on the *N-*back task with activation in the frontal lobes (predominantly prefrontal cortex) parietal lobes, and more recently, the anterior cingulate, insula, cerebellum and thalamus (see Crewther et al., 2018). Furthermore, memory span measures including the auditory forward and backward digit span tests of the WAIS (Wechsler, 2008a,b) are two of the most commonly used measures in the aging literature to examine short term and WM, respectively (Hester et al., 2004; Bopp and Verhaeghen, 2005; Elliott et al., 2011; Woods et al., 2011; Hilbert et al., 2015). Although age-related decreases in both the auditory forward and backward digit span have been reported (Salthouse, 1990; Hester et al., 2004) literature often reports that decreases in backward span are greater and more sensitive to age-related decline (Salthouse, 1990; Bopp and Verhaeghen, 2005; Elliott et al., 2011). Interestingly, in the previous studies that examined age-related decline in auditory digit span tasks, seldom have explicitly measured hearing sensitivity and ensured it was matched to controls. However, the first study to do so was conducted by Füllgrabe et al. (2015) who used a sample of audiometrically matched healthy young and older participants who were also matched on age-corrected performance IQ scores and years of education, where no significant differences were seen between older and younger participants in auditory digit span performance.

General declines in WM and cognitive processes across the lifespan are often explained by *the Processing Speed Theory of Adult Age Differences in Cognition* proposed by Salthouse (1996) which suggests that a slowing in the speed at which cognitive processing operations can be correctly executed, underlies the decline observed in higher cognitive abilities and general cognitive functions. Furthermore, decline in sensory function i.e., vision and audition that occurs in healthy aging, have been suggested to contribute to the decline in cognitive ability seen in healthy aging as suggested by theories including the *Sensory Deprivation Hypothesis*, the *Common-Cause Hypothesis*, and the *Information Degradation Hypothesis* (Lindenberger and Baltes, 1994; Baltes and Lindenberger, 1997; Schneider and Pichora-Fuller, 2000). Age group differences in cognitive functions are also commonly discussed with reference to *the Inhibitory Deficit Hypothesis* (Hasher and Zacks, 1988) which suggests that age-related declines in WM tasks are a result of older adults being less efficient in concurrently attending selectively to task-relevant stimuli and inhibiting task-irrelevant information. Furthermore, research suggests that memory span also diminishes with age, and contributes to differences seen in WM processes between young and older adults (Salthouse, 1990; Moore et al., 2005, 2006; Gazzaley et al., 2007; Mendelsohn and Larrick, 2011; Wang et al., 2011; Kirova et al., 2015; Kato et al., 2016; Vellage et al., 2016).

To date, temporal aspects of WM have mainly been discussed from the viewpoint of temporary storage and encoding of temporal stimuli, but not from the viewpoint of the functional contribution to timing and time perception *per se*. Despite this, the ability to accurately perceive and estimate time is a ubiquitous psychological process, tightly embedded with cognitive skills including attention, memory, decision making and age (Zakay et al., 1983; Casini and Macar, 1997; Block et al., 1998; Carrasco et al., 2001; Rao et al., 2001; Lewis and Miall, 2003; Tse et al., 2004; Bherer et al., 2007; Livesey et al., 2007; Perbal-Hatif, 2012; Penney et al., 2014; Turgeon et al., 2016; Baudouin et al., 2018; Polti et al., 2018). For example, research has suggested that older adults underestimate temporal interval durations both in the millisecond to second range and in the 1 min to 8 min range (Feifel, 1957; Mcgrath and O’Hanlon, 1968; Vanneste and Pouthas, 1995; Carrasco et al., 2001). However, age differences in reports of the subjective perception of time are not always consistently reported (see Friedman and Janssen, 2010). Insight from neuroimaging studies have suggested that different brain regions are reportedly responsible for time estimation in the millisecond range compared to estimating time in the seconds to minutes range (Harrington et al., 2004; Coull et al., 2011). Specifically, the basal ganglia, supplementary motor area and cerebellum have been suggested to play a role in accurate time estimation in the millisecond range (Harrington et al., 1998, 2004), whereas the prefrontal and parietal cortices, and the frontostriatal network have been suggested to underlie accurate time estimation in the second-to-minutes range (Smith et al., 2003; Wittmann, 2009). Furthermore, a recent review on the psychological and neurobiological processes associated with age-related distortions in the perception of time in the hundredths of milliseconds-to-minutes range has also suggested that age-related changes in the functioning of the cortico-thalamic-basal ganglia circuits are associated with distortions in time perception (Turgeon et al., 2016). However, Turgeon et al. (2016) have noted that older adults can still accurately estimate time by recruiting additional neural networks and cognitive resources to partially compensate for age-related declines in time perception.

Literature relating to time estimation typically explains the perception of a temporal interval either within a modular framework which suggests a specialized mechanism that is representative of the temporal relationship between events (i.e., *the Internal Clock*; Carrasco et al., 2001), or from a more general perspective, as a relationship that assumes that time perception is an intrinsic and ubiquitous property of neural activity (Ivry and Schlerf, 2008). For example, the Internal Clock Hypothesis (Carrasco et al., 2001) postulates that aging is associated with distortions to the speed of the internal clock (Craik and Hay, 1999), resulting in inaccurate time estimation for short intervals of time. Specifically, it has been suggested that compared to younger adults, the internal clock in older adults runs faster than objective time, leading to an underestimation of time intervals during experimental tasks (Mcgrath and O’Hanlon, 1968; Licht et al., 1986; Craik and Hay, 1999; Carrasco et al., 2001). Such findings support subjective reports from older adults that time seems to move more quickly compared to their younger years (Joubert, 1990). In healthy populations, debate still exists about the unifying principles that link aspects of time estimation to specific cognitive processes (Matthews and Meck, 2016). However, time estimation alongside WM tasks was recently studied with a clinical population of stroke and transient ischemic attack (TIA) patients, where residual cognitive impairment was suggested to play a role in predicting the prognosis of perceptual timing abnormalities (see Low et al., 2016).

The first study to explore time estimation in a healthy population alongside WM tasks, including the Wisconsin Card Sorting Test (WCST; Nelson, 1976), the Verbal Fluency Test (Luszcz and Lane, 2008), the Number comparison test (Babcock et al., 1997) and the Number Transformation Test (Babcock et al., 1997), was conducted by Baudouin et al. (2018). Authors aimed to examine prospective time estimation in relation to general factors corresponding to executive decline and cognitive slowing which occurs in healthy aging. The study was conducted with a sample of young (mean age = 25.8 years), old (mean age = 67.5 years) and very old adults (mean age = 82.5 years) who were required to *produce* or *reproduce* a time duration. In the production task, participants viewed a blue square on the screen, and were required to press a button when they estimated that the target duration of 5 s, 14 s or 38 s had elapsed. Alternatively, in the reproduction task, participants were asked to reproduce the exposure time for which a blue square had previously been exposed. Results revealed that information processing speed was the most reliable predictor of time estimation in the duration production task, with faster processing speed related to more accurate duration productions, while executive function was the most reliable predictor of duration reproduction, which is reported to be more reliant on WM processes.

Research into retrospective time estimation in the seconds-to-minutes range using healthy older populations’ remains relatively understudied. Thus, the current study aimed to investigate memory and processing speed performance between young and older adults of similar education, and its correlation to retrospective time estimation of a task lasting 85 s. We aimed to achieve this by using traditional measures of memory and processing speed such as visual and auditory digit span tasks, the WAIS SS and Cod, an *N*-back task using cartoon faces, a measure of retrospective time estimation, while considering affective factors (i.e., depression, anxiety and stress symptoms). The range of memory and processing speed tasks were chosen to ensure that aspects such as the requirement for rapid new learning, sustained attention and ecological relevance of tasks were all considered. In terms of age group differences, it was hypothesized that older adults would demonstrate shorter forward and backward digit spans, in line with past research (Babcock and Salthouse, 1990; Hester et al., 2004; Bopp and Verhaeghen, 2005), and a slower processing speed on the SS and Cod based on our previous results (Ebaid et al., 2017b). It was also hypothesized that older adults would perform as well as younger adults on the simple *N* = 1 back task as age-group differences are predominantly reported when *N* = 2 or more (Kato et al., 2016), but would underestimate task duration compared to younger adults. In regard to correlations, we hypothesized that retrospective time estimation would correlate to measures of memory and processing speed (Baudouin et al., 2018). However, given that there is little experimental research on retrospective time estimation of intervals in the seconds-to-minute range and its correlation to memory and processing speed tasks in healthy populations, this study is primarily exploratory in nature.

## Materials and Methods

### Participants

Ninety-nine participants comprised the sample, which included 66 young first year Psychology students from La Trobe University, Melbourne, aged between 18 and 29 who received course credit for their participation. The study also included 33 healthy older adults aged between 60 and 81, recruited from the University of the Third Age (U3A) Manningham, and received a $20 Coles-Myer voucher for their participation. U3A is an international volunteer organization where interested older individuals are able to come together and collaboratively learn, rather than for any qualifications (for more information please visit www.u3a.org.au). All participants had normal visual acuity or corrected-to-normal visual acuity, and participants who wore hearing aids were able to keep them in during the study. Though researchers ensured that vision and hearing were adequate for participants to complete all suprathreshold contrast tasks, hearing sensitivity, i.e., pure-tone or speech audiometry was not explicitly assessed. A demographic questionnaire collected information on age, gender and years of education. A measure of general negative affect: The Depression Anxiety and Stress Scale (DASS-21; Lovibond and Lovibond, 1995) was administered as a screening tool. Exclusion criteria included previous diagnoses of a neurological disorder or inability to speak or understand English with basic competence, though no participant was excluded on this basis. The demographic information of the sample is summarized in Table 1.

**Table 1:** Characteristics (means and standard deviations) of participants.

	Young adults *N* = 66		Old adults *N* = 33	
	*M*	*SD*	*M*	*SD*
Age (years)	19.60	2.26	70.30	5.62
Gender (M/F)	8/59		8/25	
Education (years)	7.64	1.05	10.91	3.38
Depression score (DASS-21)	3.09	2.51	1.79	1.93
Anxiety score (DASS-21)	3.34	3.03	3.91	2.88
Stress score (DASS-21)	5.89	3.49	1.79	1.93

*Note: years of formal education was recorded from the first year of secondary school onwards, i.e., completion of secondary school was recorded as 6 years of completed formal education. Maximum score on each subset of the Depression Anxiety and Stress Scale-21 (DASS-21; i.e., depression, anxiety, stress) was 21, with higher scores indicating more severe symptoms*.

## Memory Measures

### Forward and Backward Digit Span (Auditory and Visual)

The forward and backward digit span tasks were adapted from the Auditory Digit Span subset of the WAIS 4th Edition (Wechsler, 2008b). A customized digit span task made using Authorware Professional software was administered auditorily and visually on an Apple iMac (Retina 4K) computer with a 21.5-inch monitor. The forward digit span task reportedly measures attention in the context of immediate recall of information from short-term memory, while the backward span places demands on both attention and WM abilities in requiring manipulation (i.e., reordering) of the information (Lichtenberger and Kaufman, 2009). The forward digit span task involves presenting participants with a series of random digits (from 1 to 9) after which participants are required to repeat the list back in the same order either verbally, or by typing their response using a keyboard, as preferred. If successful, the participant is given a longer sequence. In the backwards digit span condition, participants are presented with a series of random digits (from 1 to 9), and are required to repeat the list backwards, again either verbally, or by typing their response using a keyboard, as preferred. The number sequence began at a span length of two, and increased by one after two correct trials, i.e., participants were given two trials per span length. Once two trials of the same span length were answered incorrectly, testing was discontinued, and the previously correct span length was recorded as the participants “digit span capacity.”

The task always began with the forward digit span task followed by the backward digit span, and the order of presentation between the visual and auditory conditions were counterbalanced across participants (counterbalancing of tasks is later described). Prior to commencing the task, participants were seated approximately 60 cm away from the computer screen and the researcher verbally explained the instructions of each digit span task to participants. In the *visual* digit span condition, digits were presented in black Ariel 92pt font against a white background, at a rate of one digit per second, with no sound/voiceover reading the numbers. In the *auditory* condition, digits were read aloud i.e., verbally presented to participants via a voice over on loudspeaker on the Apple iMac computer as part of our custom modified computer task, with no visual representation of the numbers on the screen. After each number sequence, the following words appeared on the screen: “*Please type your response”* in black Ariel 48pt font, prompting participants to repeat the digit sequence. The next trial only commenced once the *Enter* key was pressed on the keyboard. As part of the computerized digit span task, verbal instructions were also provided to participants via voice-over iterating the task requirements for the forward and backwards digit span immediately before commencement. For example, for the visual forward digit span, the voice over script was as follows: “*You are going to see some numbers. After each trial, you will have to repeat these numbers in the same order. Ready?”* Participants were then required to press the space bar to begin the task, or had the option to replay the instructions. The computerized instructions and the auditory presentation of numbers were played via loudspeaker on the Apple iMac computer in which the volume was set to a level preferred by the participant. This was adjusted during the computerized instructions phase, prior to formal commencement of the task.

#### N-Back

A modified version of the *N*-Back task originally developed by Kirchner (1958) was administered to participants on a computer, as a simple measure of sustained attention and WM, and took a total of 85 s. Participants were presented with a sequence of 34 cartoon faces that varied in shape (i.e., 9-star shaped faces, 9-round shaped faces, 8-sqaure shaped faces and 8-oval shaped faces) at a rate of 1.5 s per stimulus, with a 1 s delay between stimuli. Participants were required to indicate by pressing the space bar, if the current image was the same as the image presented immediately prior (i.e., *N* = 1 back). This was the case on seven occasions, and so the maximum correct score participants could obtain was 7. An *N* = 1 back was chosen on the basis of the task demands being relatively simple for both age groups as stimuli were presented for adequate time for participants to encode. In doing so, we aimed to ensure both groups of participants’ sustained attention for the full duration of task in order to provide a true measure of time estimation at completion.

### Processing Speed Measures

#### Symbol Search (SS) and Coding (Cod) (WAIS-IV)

As per the WAIS-IV kit instructions, the SS and Cod subtests from the WAIS-IV were administered for 2 min each. During SS, two target symbols appearing on the left of a row are sought among an array of five symbols on the right. The individual responds by marking with a pencil either the identical symbol, or a “no” box (if the matching symbol is not present in the array). Performance was measured as the number of symbols accurately identified in 2 min, with more symbols accurately completed indicative of faster processing speed. In healthy adults, raw scores on SS have been shown to decline by more than 50% between the ages of 25 and 65 (Wechsler, 2008b).

The Cod task requires an individual to copy the appropriate symbol in a box underneath a digit (one-to-nine), while referring to a key at the top of the page containing digits and their corresponding symbols. Performance is based on the number of pairs correctly copied in 2 min, with more pairs correctly copied indicative of a faster processing speed.

### Time Estimation

At the end of the *N*-back task participants were asked “*how long do you think that task took in seconds?”* and their estimation was recorded, in turn assessing retrospective time estimation. No prior warning was given to participants about having to estimate the task duration, in order to prevent any strategies of time-keeping or monitoring of temporal information and thus, simulating a more realistic example of “on the spot” retrospective time estimation. Examining retrospective time estimation with no prior warning can only be done once in order to obtain a genuine estimate from participants. Though this may impede on the reliability of the data, if participants are asked to estimate time of several tasks, this data is unlikely to reflect a pure measure of retrospective time estimation without prior warning. This method of retrospective time estimation using the same *N*-back task has been used in recent research with a healthy and clinical sample of stroke and TIA patients (though task conditions were *N* = 0; see Low et al., 2016).

### Procedure

All participants were guided through the experimental tasks. To counterbalance the order of presentation of tasks between participants, Microsoft Excel was used to generate all the possible arrangements of the following number sequence (1, 2, 3, 4, 5) which represented the auditory digit span (forward and backward), the visual digit span (forward and backward), SS, Cod and the *N*-back with time estimation, respectively. This generated a total of 120 arrangements in which the first 66 sequences were used for the young participants, and the first 33 sequences were used for the older participants. All testing was conducted in a quiet room either at La Trobe University or U3A, where only the participant and experimenter were present. All except the time estimation task were preceded by practice trials and total testing time took approximately 1 h.

### Data Analysis

All analyses were performed using SPSS v. 25.0 (IBM Corp., Armonk, NY, USA). Data was screened for outliers on individual tasks and for any result indicating inconsistent performance across tasks. None were found. Bonferroni adjusted alpha levels of 0.0125 (0.05/4) per digit span test and 0.025 (0.05/2) per SS and Cod were applied to correct for multiple comparisons for the *t*-test and correlational analyses.

## Results

Means and standard deviations were calculated for performance on all dependent measures for young and older adults. These results are presented in Table 2.

**Table 2:** Descriptive statistics and independent samples *t-test* for mean difference on measures of memory, processing speed and retrospective time estimation in young and older adults.

	Young adults				Older adults				Age-group differences on measures	
Measure	*N*	Range	*M*	*SD*	*N*	Range	*M*	*SD*	*p*	*η*^2^
DS.Aud.FS	66	4–10	6.91	1.10	33	3–9	6.09	1.23	0.001**	0.330
DS.Aud.BS	66	3–8	5.88	1.08	33	3–7	5.06	1.12	0.001**	0.279
DS.Vis.FS	66	4–9	6.56	1.15	31	4–11	5.87	1.45	0.013*	0.242
DS.Vis.BS	66	3–8	5.72	1.23	31	2–7	5.23	1.28	0.069	0.241
*N*-back	66	4–7	6.92	0.62	33	5–7	6.85	0.83	0.422	0.319
SS	66	24–60	39.54	7.69	33	16–48	28.42	7.05	<0.001**	0.549
Cod	66	37–121	78.73	13.09	33	31–91	53.55	12.59	<0.001**	0.575
Time Est (s)	65	15.00–120.00	46.20	22.00	33	10.00–120.00	42.73	25.44	0.486	0.279

Note: DS.Aud.FS, Auditory Forward Digit Span; DS.Aud.BS, Auditory Backward Digit Span; DS.Vis.FS, Visual Forward Digit Span; DS.Vis.BS, Visual Backward Digit Span; SS, Symbol Search; Cod, Coding; Time.Est, Retrospective Time Estimation; i.e., Estimated duration of N-back task. **p < 0.01, *p < 0.05 (two-tailed, Bonferroni correction).

## Relationships Between Measures of Memory, Processing Speed, Retrospective Time Estimation and Age

Correlational analyses on the entire sample were performed to investigate the strength, direction and significance of associations between measures of memory, processing speed, retrospective time estimation, and age. As our age distribution was not normally distributed and included two distinct age groups, correlational analyses using Spearman’s Rank-Order Correlation was used to examine this.

Results revealed no significant correlation between retrospective time estimation and any measure of memory or processing speed. Furthermore, there were no significant correlations between age and time estimation (*r*_s_ = −0.189). However, significant correlations were demonstrated between age, memory spans and speed performance, with shorter auditory and visual spans and slower speed performance associated with older age. Specifically, shorter auditory forward and backward span was weakly but significantly associated with older age (*r*_s_ = −0.223, *r*_s_ = −0.203, respectively). Furthermore, shorter visual forward span was weakly but significantly correlated with older age (*r*_s_ = −0.213), and slower performance on the SS and Cod was significantly moderately correlated with older age (*r*_s_ = −0.608, *r*_s_ = −0.607, respectively). A full correlation table of age and all dependent measures is shown in Table 3.

**Table 3:** Spearman’s rank-order correlations between measures of memory, processing speed and retrospective time estimation.

Measure	Age	DS.Aud FS	DS.Aud BS	DS.Vis FS	DS.Vis BS	*N*-back	SS	Cod	Time Est (s)
Age	-							
DS.Aud.FS	−0.223*	-							
DS.Aud.BS	−0.203*	0.410*	-						
DS.Vis.FS	−0.213*	0.334**	0.316**	-					
DS.Vis.BS	−0.125	0.250*	0.361**	0.347**	-				
*N*-Back	−0.121	0.205*	0.239*	0.156	0.043	-			
SS	−0.608**	0.122	0.171	0.084	0.213*	0.041	-		
Cod	−0.607**	0.229*	0.380**	0.258*	0.215*	0.106	0.657**	-	
Time Est (s)	−0.189	0.065	0.070	0.187	0.144	−0.018	0.038	0.124	-

Note: DS.Aud.FS, Auditory Forward Digit Span; DS.Aud.BS, Auditory Backward Digit Span; DS.Vis.FS, Visual Forward Digit Span; DS.Vis.BS, Visual Backward Digit Span; SS, Symbol Search; Cod, Coding; Time.Est, Retrospective Time Estimation; i.e., Estimated duration of N-back task. **p < *0*.01, *p < 0.05 (two-tailed, Bonferroni correction).

## Age-Group Differences in Performance on Measures of Memory, Processing Speed and Retrospective Time Estimation

An independent-samples *t*-test was conducted to compare performance on measures of memory, processing speed, and retrospective time estimation between younger and older adults. Significant differences in performance between age groups were demonstrated on the auditory but not the visual digit span tasks, with younger adults demonstrating a significantly longer memory span compared to older adults on the auditory spans. Specifically, younger adults had a significantly larger auditory forward span compared to older adults (*p* < 0.01, *η*^2^ = 0.330), but no significant differences were demonstrated in the visual forward span compared to older adults (*p* < 0.05, *η*^2^ = 0.013). Furthermore, younger adults had a significantly larger auditory backward span compared to older adults (*p* < 0.01, *η*^2^ = 0.279), though no significant difference between age groups was demonstrated on the visual backwards span (*p* = 0.069, *η*^2^ = 0.241).

Results also revealed significant differences on the SS and Cod where younger adults demonstrated a faster processing speed compared to older adults (*p* < 0.01, *η*^2^ = 0.549, *p* < 0.01, *η*^2^ = 0.575, respectively). No significant differences between age groups were demonstrated on the *N*-back task in terms of accuracy (*p* = 0.422, *η*^2^ = 0.319) or in retrospective time estimation (*p* = 0.486, *η*^2^ = 0.279). These results are presented in Table 2.

## Discussion

The aims of the current study were to assess performance on memory and processing speed measures in healthy young and older adults, and to investigate time estimation in a more novel way, from the viewpoint of retrospective time estimation of a short temporal interval of 85 s. The current study also aimed to explore how performance on memory and processing speed measures correlate to retrospective time estimation. Age group differences in performance on memory, processing speed and time estimation will first be discussed, followed by the relationship between memory and processing speed tasks with retrospective time estimation.

### Age Group Differences in Memory, Processing Speed and Time Estimation

Consistent with hypotheses and past research, memory span (forward and backward) as measured by the auditory digit span task was shorter in older adults compared to younger adults (Wechsler and De Lemos, 1981; Craik et al., 1990; Foos and Wright, 1992; Bopp and Verhaeghen, 2005; Elliott et al., 2011). However, memory span as measured by the visual digit span, was not significantly different between young and older adults, which was not in line with hypotheses and past research (Craik et al., 1990; Foos and Wright, 1992).Previous research often reports larger age-related effects for auditory backward memory span compared to forward memory spans (Craik et al., 1990; Foos and Wright, 1992; Bopp and Verhaeghen, 2005), which is not entirely reflective of results from the current study, with age differences seen in both the auditory forward and backward digit spans. It is important to note however, that although the older adults in the current study had normal or corrected-to-normal vision, they were not explicitly assessed for optimal hearing and auditory deficits. In addition, our older sample also had an average of three extra years of formal education compared to younger adults, and these two factors may provide an explanation for the comparable performance on the visual digit span task but the difference in performance on the auditory digit span. Indeed, higher levels of education have been suggested to “lower the load” of cognitive tasks by conferring a greater ability to activate appropriate neural networks, and thus improve task performance (Archer et al., 2018). Furthermore, from the viewpoint of theories which postulate an association between sensory and cognitive decline (Lindenberger and Baltes, 1994; Baltes and Lindenberger, 1997; Schneider and Pichora-Fuller, 2000), it is possible that sensory loss associated with the normal aging process contributed to the older adults having a shorter span on the auditory digit span tasks in the current study. More specifically, there may have been an indirect effect of uncorrected age-related hearing loss during the auditory digit span tasks which was not explicitly accounted for in the current study. Indeed, recent research reported no age group differences on the auditory forward and backward digit span tasks in a sample of healthy young and older participants who were matched on age-corrected performance IQ scores, years of education and were also audiometrically matched (Füllgrabe et al., 2015).

Processing speed as measured with the WAIS SS and Cod was significantly different between groups, with older adults demonstrating a slower speed, in comparison to younger adults. This was in line with hypotheses and past research (Hoyer et al., 2004; Gilmore et al., 2006) especially with regard to the SS and Cod which are WAIS clinical measures of processing speed, that are also heavily reliant on motor speed (Ebaid et al., 2017b). In line with this, manual motor speed has been consistently reported to slow with increased age (Murata et al., 2010; Ebaid et al., 2017b). Elevated depression and anxiety symptoms are also often reported to impede cognitive performance in older populations (Beaudreau and O’Hara, 2008, 2009), however, it is interesting to note that our healthy older sample reported lower depression anxiety and stress scores than our younger adults, making negative affective issues unlikely to have impeded task performance in this case. As expected, scores on the *N*-back task were not significantly different between young and older adults, with both groups obtaining almost perfect total scores i.e., accurately detecting all seven cases where the current image was the same as the image presented prior. As we have previously shown that threshold exposure time for healthy older adults to accurately identify visual stimuli is approximately 136 ms and 890 ms to detect change between two visual arrays (Ebaid et al., 2017a), ceiling performance by both groups on the *N*-back was not a surprising finding.

Retrospective time estimation of the *N*-back task was not significantly different between young and older adults, with both groups underestimating the duration of the task, which was contrary to predictions. On average, older adults estimated that the task lasted 42 s, and younger adults estimated 46 s. As both groups substantially underestimated task duration, these results contradict past research which report that only older adults underestimate time (Craik and Hay, 1999; Carrasco et al., 2001) and as such, these results do not add credence to the *Internal Clock Hypothesis*. It is important to note that these earlier studies which explored age differences in interval time estimation made their participants aware prior to the task that they would have to estimate time. In doing so, these studies examined *prospective time estimation* and thus, participants could potentially consciously monitor time. In the current study, we examined *retrospective time estimation*, and participants were not given prior warning that they would have to estimate task duration, which may explain the discrepancy in findings between past and current research. In past research that reported younger adults demonstrating an overestimation or *lengthening effect* of reproduced temporal intervals, it has been attributed to the monotony of the task and decreased arousal due to boredom (Treisman, 1963; Hicks and Allen, 1979), and thus, the time interval was thought to be perceived as longer than objective time. The potential for mood and affective states such as boredom to influence the perception of time dates back to the early 1890s where William James wrote “*our feeling of time harmonizes with different mental moods”* (James, 1892), and is also implied in commonly understood phrases such as “*a watched pot never boils,”* or “*time flies when you’re having fun*.” As participants in the current study obtained almost 100% accuracy on the *N*-back task, it is conceivable that they remained engaged, making it unlikely that their estimation of time was confounded by boredom or monotony of task. It may be the case then, that participants found the *N-*back task easy and/or rewarding, thus requiring less cognitive effort, and in turn perceiving the task as taking a shorter time. Indeed, the subjective duration of a task has been reported to be influenced by the amount of information processing resources required for accurate performance on the task (see Brown, 1997 for a review) with increased task complexity reported to lead to the increased perception of a temporal interval (Macar et al., 1994; Penney et al., 2014). Such reports may also be interpreted in the context of the *neural efficiency hypothesis* (Haier et al., 1988), in that if participants dedicated less neural resources to a task (suggesting more efficient processing), it is likely that estimates of task duration will be shorter than objective task duration. Interestingly, our time estimate results also contradict those found in Low et al. (2016), who measured retrospective time estimation of a similar, but easier *N*-back task, where *N =* 0 i.e., participants were required to respond each time they saw a target stimulus instead of responding when the current image matched the one presented one stimulus earlier (as used in the current study). This was assessed in a clinical sample of stroke and TIA patients, and healthy controls (mean age = 55 years) in which the healthy controls were estimating that the task took an average of 86 s, which is strikingly close to actual task duration. However, the healthy participants in Low et al. (2016) may not be comparable to those from the current study, as requirement for participation was not comparable education to a university population nor were they continuing to engage in vocational education, as in the current study. Furthermore, participants in Low et al. (2016) were predominantly kin to the patients or recruited in response to flyers at the hospital from where the clinical sample was derived, and so it may be reasonable to assume that they were tested in circumstances that differed substantially from the current study, i.e., following their relative recently suffering a stroke or TIA. These factors may indicate that the two samples, though both neurologically healthy, are not comparable in other domains and thus, may play a part in the discrepancy in findings between studies.

### The Relationship Between Memory and Processing Speed With Retrospective Time Estimation

Contrary to hypotheses, there were no significant relationships between memory and processing speed with retrospective time estimation. This also contradicts past research which demonstrated that a faster processing speed was correlated to more accurate “duration productions” while more efficient executive function (in particular, set shifting and cognitive flexibility) as measured by the WCST (Nelson, 1976) was the most reliable predictor of “duration reproduction” (Baudouin et al., 2018). In the current study, we chose to use memory measures including the backward digit span and SS and Cod as correlative measures with retrospective time estimation, and thus, the difference in tasks may also explain the difference in findings between the current study and Baudouin et al. (2018). Further, it is important to note that the discrepancy between results from the current study and past research by Baudouin et al. (2018) is at least partly due to our study measuring retrospective time estimation, and not prospective time estimation. Indeed, it has been suggested that these two aspects of temporal estimation require different cognitive resources (Block et al., 1998; Pouthas and Perbal, 2004; Zakay and Block, 2004) with prospective time estimation suggested to rely on attentional resources and the ability to divide attention between the task and temporal information in order to keep track of time (Pouthas and Perbal, 2004), whereas retrospective time estimation or *remembered duration* is suggested to be a function of the amount of memory storage space available for events that occurred during a particular interval of time (Block, 2014). With this in mind, our results do not support the suggestion that accurate retrospective time estimation is reliant on implicit memory storage, with no significant correlation found between retrospective time estimation and any explicit memory span task. A recent study conducted by Polti et al. (2018) with 24 healthy young adults also demonstrated the effect of attention and WM load during four *N*-back conditions (i.e., 0, 1, 2 and 3 back) on prospective time estimation. Results showed that paying attention to time duration lengthens subjective duration and that dividing attention between monitoring time and concurrent WM task shortens perceived duration. However, these findings were not replicated in the current study.

### Limitations

The generalizability of our study is limited in relation to other populations who may not have comparable education levels in both young and older samples. This is particularly the case for older adults where years of education is reported to potentially protect against cognitive decline and forms of dementia in later years in life (Zhang et al., 1990; Armstrong et al., 2012). Specifically, the older adults who were recruited from U3A have continued to engage in vocational study post-retirement, and thus, may not be representative of the general population of older adults over 60. In addition, given that we did not conduct explicit audiometric screens for our older participants, it may be the case that deficits in auditory processing impacted the results, given the prevelence of deficits in auditory processing that occurs with normal aging (Lindenberger and Baltes, 1994; Baltes and Lindenberger, 1997). Thus, future research should aim to include audiometric screening tests when conducting similar cognitive research. Furthermore, as retrospective time estimation of temporal intervals in the seconds to minutes range remains an understudied area of research with healthy older populations, it may be useful to further examine using several conditions where task demands and complexity are manipulated, and in more ecologically valid contexts.

## Conclusions and Future Directions

The current study extended findings arising from cognitive aging literature, in that auditory memory span and processing speed tasks decline with age, but not in conditions with decreased cognitive load i.e., in our *N*-back condition where *N* was equal to 1 (Kato et al., 2016), and stimuli were presented for adequate durations to allow encoding and memory. These results should inform areas of cognitive aging research with a particular focus on the aspects of cognitive processing that may be susceptible to aging, and which aspects may remain intact in a healthy older population. Our study was novel in examining retrospective time estimation of a relatively short temporal duration (85 s), with educated healthy older adults, comparable to our younger sample of university students. Our participants were not pre-warned that they would be asked to reflect and estimate the duration of a recent time interval, and so our results are unique in providing preliminary insight into retrospective time estimation, bearing in mind that the task loses validity if participants are asked to estimate time on several occasions. We showed that both young and older populations substantially underestimated time when asked to estimate the duration of a simple task when no prior warning was given before commencement of the task. Our study revealed no significant correlations between memory and processing speed, and retrospective time estimation which differs substantially from previous research into estimation of a temporal interval and healthy aging. Future research is needed to uncover the memory processes that are unique to accurately estimating time in this manner, and the factors that may be contributing to such underestimations of time.

## Ethics Statement

This study was carried out in accordance with the recommendations of the National Statement on Ethical Conduct in Human Research, La Trobe University Human Ethics Committee (UHEC), with written informed consent from all subjects. All subjects gave written informed consent in accordance with the Declaration of Helsinki. The protocol was approved by La Trobe UHEC, approval number S15/19.

## Author Contributions

The study was initiated by SC who also designed the outline and content. DE recruited participants and collected the data. Both DE and SC analyzed the data and contributed to the writing.

## Conflict of Interest Statement

The authors declare that the research was conducted in the absence of any commercial or financial relationships that could be construed as a potential conflict of interest.
